# Population density and temperature correlate with long-term trends in somatic growth rates and maturation schedules of herring and sprat

**DOI:** 10.1371/journal.pone.0212176

**Published:** 2019-03-06

**Authors:** Aidan Hunter, Douglas C. Speirs, Michael R. Heath

**Affiliations:** Marine Population Modelling Group, Department of Mathematics and Statistics, University of Strathclyde, Glasgow, United Kingdom; Technical University of Denmark, DENMARK

## Abstract

We examine long-term trends in the average growth rates and maturation schedules of herring and sprat populations using survey data collected from the North Sea and west of Scotland since the 1960s and 1980s respectively. Otolith age data and maturity data are used to calculate time series of mean lengths at age, von Bertalanffy growth parameters, and probabilistic maturation reaction norms. As the growth and maturation of fish is known to be influenced by temperature and stock abundances, we account for these variables using Generalised Additive Models. Each of the herring populations displayed either steady declines in mean length across multiple age groups, or declines in length followed years later by some recovery. Depending on region, lengths at age of sprat increased or decreased over time. Varying temporal trends in maturation propensity at age and length were observed across herring populations. Many of the trends in growth rate and maturation were correlated to population abundance and/or temperature. In general, abundance is shown to be negatively correlated to growth rates in herring and sprat, and positively correlated with maturation propensity in herring. Temperature is also shown to be correlated to growth and maturation, and although the effect is consistent within species, the temperature effects differ between herring and sprat. This study provides detailed information about long-term trends in growth and maturation, which is lacking for some of these pelagic stocks, especially in the west of Scotland.

## Introduction

Marine fish species worldwide are generally getting smaller [1], and many exploited populations are maturing at increasingly young ages and small sizes [2]. As these trends in growth rates and maturation schedules have occurred worldwide, in species with a range of physiological and behavioural characteristics, they are most likely responses to pervasive phenomena such as warming temperatures and fishing [3, 4]. Rising temperatures are linked to declines in the growth rates of ectotherms [5, 6], and have been predicted to cause a 14%–24% decrease to the average maximum weight of marine fish between 2000 and 2050 [1]. Fishing may influence growth or maturation through a range of mechanisms including density-dependent effects, relaxation of predation pressures, and selection on phenotypes [7]. There is concern that size-selective fisheries reduce genetic diversity of harvested species by selecting for slow growth, small maximum sizes, and in particular, early maturation [3, 8]. In contrast to plastic responses to environmental variations, any reversals of evolutionary trends in growth and maturation are likely to occur slowly over generations [9]. Whether plastic or evolutionary, changes in growth rates and maturation schedules may have a range of consequences for ecosystems and fisheries. Declines in growth rates and maximum sizes reduce the potential yield of fisheries [3], and may influence predator-prey interactions [10, 11]. Early maturation, combined with high mortality rates of old, large fish, may reduce the reproductive potential of stocks and their ability to recover from depletion [12, 13]. Moreover, increasingly early maturation has been postulated to indicate potential stock collapse [14]. This means that retaining large, hence highly fecund [15], individuals within stocks may be crucial to ensuring the long-term sustainability of fisheries [13, 16]. Knowledge of trends in growth rates and maturation schedules may therefore help inform policy for fishing strategies and ecosystem management.

Previous studies have reported increasingly early maturation and declines in size of several North Sea and west of Scotland fish stocks. A study of a North Sea community found declines in maximum size, synchronous with an increase in temperature, in six out of the eight species considered [17]. A probabilistic maturation reaction norm (PMRN) analysis demonstrated that trends of increasingly early maturation in North Sea haddock (*Melanogrammus aeglefinus*), whiting (*Merlangius merlangus*), and male cod (*Gadus morhua*) were consistent with fisheries-induced evolution, whereas earlier maturation of female cod was related to rising temperature [18]. Several west of Scotland demersal stocks have also exhibited declines in growth rates, maximum sizes, and lengths at maturation [19, 20]. Few of these declines were attributable to trends in temperature or abundance, and it was suggested that they may have resulted from decades of size-selective trawl fishing. Most studies of long-term changes in the growth and maturation of fish populations, including those of the North Sea and west of Scotland, have focused on demersal species. With the exception of herring (*Clupea harengus*), pelagic species tend to receive relatively little attention. Previous studies of trends in the growth and/or maturation of herring and sprat (*Sprattus sprattus*) include: comparing the growth of 15 North Atlantic herring populations and relating growth to temperature and population density [21]; analysing long-term temporal trends in the maturation schedules and lengths at age of Norwegian herring [22]; examining factors influencing the growth of Baltic Sea sprat [23]; and numerous International Council for the Exploration of the Sea (ICES) reports e.g. [24]. In general, changes to the growth and maturation of these pelagic species appear to be plastic responses to trends in environmental factors, often temperature or abundance.

**Fig 1:**
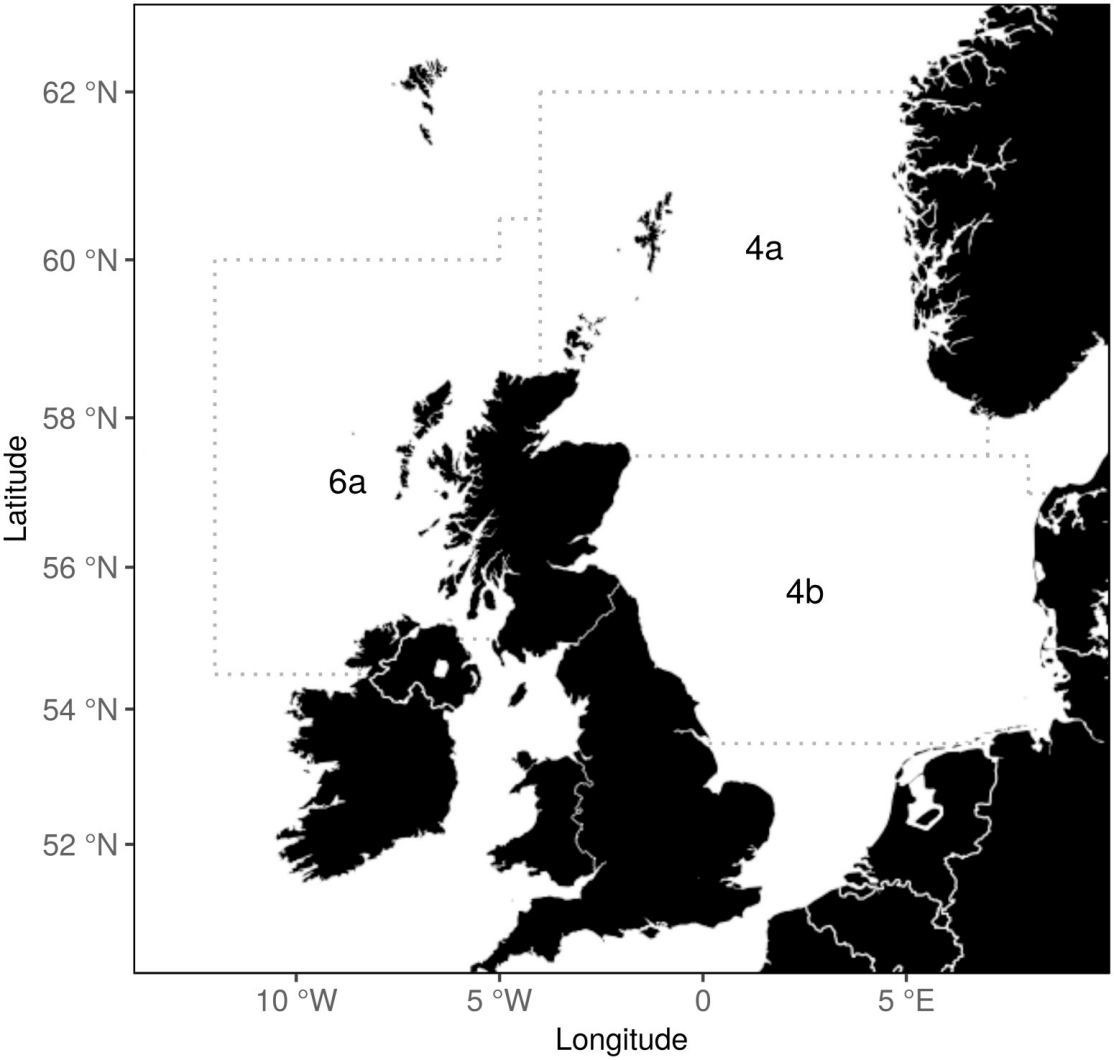
The three ICES fishing areas considered in this study.

We use time series of bottom trawl survey data, collected from the North Sea and west of Scotland since the 1960s and 1980s respectively, to investigate trends in average lengths at age, and ages and lengths at first maturation of herring and sprat. Long-running time series of acoustic survey data would be more appropriate for these analyses as these are pelagic species. However, as such data were not available we take advantage of bottom trawl data acknowledging that it may not be fully representative of pelagic stocks. We grouped the data by ICES fishing areas to independently examine the growth and maturation of fish sampled from different environments (Fig 1).

Our aims in this study are to determine long-term trends in the growth rates and maturation schedules of herring and sprat, and to assess whether these trends are related to variation in temperature and stock abundances. We derive age-length distributions and time series of mean lengths at age, then fit von Bertalanffy growth curves [25] to each cohort. The von Bertalanffy growth parameters, namely, asymptotic length, *L*_∞_, von Bertalanffy growth rate, *k*, and length at age zero (initial length) *L*_0_, are used to derive trends in age-specific growth rates. Trends in age and length dependent maturation schedules are found by estimating PMRNs for each cohort [26]. We then examine how growth rates and maturation have been influenced by variation in temperature and stock abundance by including these variables in Generalised Additive Models (GAMs) predicting growth rates at age and maturation probabilities.

## Materials and methods

### Data

The principal data source were the International Bottom Trawl Surveys (IBTS), conducted by all participating nations, for the North Sea (areas 4a and 4b) and Scottish west coast (area 6a) [27]. Detailed information on this survey is given in [28]. Herring trawls were the main gear used by the IBTS during the late 1960s and 1970s. As the focus of the IBTS broadened from just herring, the more multi-purpose Grande Overture Verticale trawl gear became the standard in 1983. The duration of trawls was 1 hour, until 1978 when this was reduced to 30 minutes by all participating nations except Scotland, which did not reduce trawl duration until 1998. The IBTS is spatially stratified by ICES statistical rectangles of approximately 1 degree longitude × 0.5 degree latitude, and typically each rectangle is trawled at least twice per year. We extended the data set backwards in time, where possible, using survey samples collected by Fisheries Research Services (FRS). These data included bottom trawl samples from several surveys including the IBTS, with some samples pre-dating the IBTS time series. Besides extending the time series data, the FRS data were also useful for distinguishing between autumn and spring spawning herring. Although the sampling protocols will have differed from the IBTS, which used standardised gear from 1983, the FRS data provide valuable information on historic age-length structure and maturity.

We grouped the survey data by ICES fishing areas 4a, 4b, and 6a (Fig 1). North Sea (areas 4a and 4b) herring form four main stocks based on spawning location: the Orkney and Shetland stock, the Buchan stock around Aberdeen bank, the Banks stock along the English east coast, and the Downs stock in the English channel [29]. Apart from the winter spawning Downs stock, North Sea herring spawn in autumn, from August to October [30]. These stocks mix throughout the North Sea at other times of the year. Spring spawning herring from the Skagerrak and Kattegat seas and the west coast of Norway may also mix with the North Sea autumn spawners [31]. As North Sea herring are assessed as a single stock and the survey data does not distinguish the various sub stocks, we group the data by areas 4a and 4b to represent herring living in the northern and southern North Sea; acknowledging that variation in the relative abundance of sub stocks may influence the perception of growth and maturation trends. The western herring stock is west of the British Isles, and currently managed as two sub stocks defined by spawning location and timing [32]. However, individuals spawned by one population may recruit to neighbouring populations [33], and otolith microchemistry analysis suggests that the western herring are essentially one population with discrete spawning locations and timings [34]. Herring from the western stock are also found in the North Sea [30, 35], as they can be transported there by the northerly Atlantic current as larvae [36, 37]. Individuals may then migrate back from the North Sea to spawn in their natal breeding sites in the west of Scotland [38]. We consider the survey data collected from area 6a as representing the entire western stock, recognising this simplification does not account for variation in the abundance and distribution of sub stocks. We did some additional analyses, however, examining trends in the mean length at age of herring sampled from the Firth of Clyde, finding significant long-term declines, but also finding this had negligible impact on inferences about herring growth in the wider 6a area.

North Sea sprat are most abundant from the English east coast to the Southern Bight and the west coast of Denmark, and are also numerous in the Moray Firth [39]. Sprat spawn offshore in open waters from March until August, depending on temperature, with peak spawning during May and June [40]. Larvae drift inshore into estuaries and sea-lochs which become nurseries for juveniles [41]. Despite some evidence suggesting sprat populations from the German Bight, Kattegat, and Celtic Sea are genetically distinct [42], there is little detailed information about the stock structure and potential migrations of North Sea sprat, which are assessed as a single stock [24]. Sprat live all around the British and Irish coasts [43], and less is known about the stock structure of the western population. Spawning occurs in spring and early summer [44]; currents transport larvae to estuarine and sea-loch nursery grounds [45]; then after growing sufficiently they migrate to deeper waters. Western sprat are assessed over much of the Celtic Sea (ICES areas 6 and 7), and most sprat fisheries here are sporadic, occurring in various locations at different times of year [24]. As with the herring data, we grouped the sprat survey samples by ICES areas 4a, 4b, and 6a.

We used data from a single quarter of the year within each separate analysis. Quarter 1 samples were used exclusively for the growth analyses because these were the longest time series and had the fewest years with no records. The time span of the data sets depended on region, species, and quarter of the year. The longest running time series were herring samples collected from area 4b during quarter 1, spanning 1966–2012.

The data sets comprised information on length, age, and maturity. The length of each fish in a haul is measured and rounded down to the nearest 0.5*cm*. The ages and maturity status of length stratified sub samples are determined by examining otoliths and gonads. The mean size and the range of the annual samples for length, age, and maturity for each species and region are shown in Table 1. Ages were recorded as the observed number of winter otolith rings, so the assumed ‘birthdays’ of fish were the January 1^st^ nearest to spawning date. We use ‘age group’ instead of ‘age’ to refer to the recorded ages of fish because of these discrepancies between the recorded and true ages. The maturity data contain information on the sex and maturity status of each individual, determined by visual inspection of the gonads. FRS samples used a more detailed maturity status scale than the IBTS. The IBTS maturity scale was: (1) immature, (2) maturing, (3) spawning, and (4) spent. The FRS maturity scale was: (1) juvenile, (2) virgin-maturing, (3–5) maturing, (6) spawning, (7) spent, and (8) resting. We considered stages (2–5) and (7–8) on the FRS maturity scale as equivalent to stages (2) and (4) on the IBTS scale. Observation error associated with examining otoliths and gonads was not reported in the data sets, and the potential for observation error-induced biases were not considered in this study.

**Table 1:** The mean number of fish sampled for length, age, and maturity status each year, with the range shown in parenthesis. Years without data have been excluded.

Species	Region	Quarter	Length	Age	Maturity
Herring	4a	1	59483 (2–507936)	743 (2–2304)	647 (1–1938)
Herring	4a	3	58613 (886–284888)	944 (234–1936)	972 (133–1733)
Herring	4b	1	288023 (9378–948674)	944 (234–1936)	972 (133–1733)
Herring	4b	3	221369 (979–677919)	1217 (172–2845)	1071 (113–2099)
Herring	6a	1	76911 (7213–330262)	1310 (187–2040)	1295 (181–2040)
Sprat	4a	1	17714 (30–156346)	122 (6–277)	111 (6–206)
Sprat	4b	1	332975 (3663–1187217)	763 (16–2840)	434 (3–894)
Sprat	6a	1	9222 (208–23177)	215 (1–524)	

It is preferable to study maturation using data collected during, or just prior to, the spawning season because this is when gonads reach a state of maturity. We therefore used maturity data from quarters 1 and 3 to analyse the maturation of spring and autumn spawning herring. Unfortunately autumn spawning herring from area 6a and spring spawners from area 4b had to be analysed using data from quarters 1 and 3 respectively. In area 6a, this was because survey data were collected in quarters 1 and 4, and only data collected during quarters 1 or 3 should be used to distinguish between spring and autumn spawning herring [46]. Whereas in area 4b, although maturity data were available in quarters 1 and 3, the IBTS data were available in all of the years covered by the FRS data for quarter 1. This prevented categorizing herring as spring or autumn spawners due to the less informative IBTS maturity scale. See S1 Appendix for full details of how autumn and spring spawning herring were distinguished using the maturity data.

The herring abundance data used were North Sea and west of Scotland stock assessment estimates of total stock biomass [24]. It should be noted that these abundance estimates are dependent upon assumptions regarding the natural mortality scheme used by the assessments. In this case the natural mortality rates, derived from multispecies modelling of the North Sea [47], vary over time and age group, and consisted of model-estimated predation rates and some chosen base mortality rate. As the assessment of western sprat covers an area far wider than 6a, these estimates of abundance were not suitable for this study. Annual relative abundance estimates for sprat were derived by dividing the number of sampled individuals by the total area swept out by the survey trawl gear each year. For consistency we used these survey-derived relative abundance indices for western and North Sea sprat.

**Fig 2:**
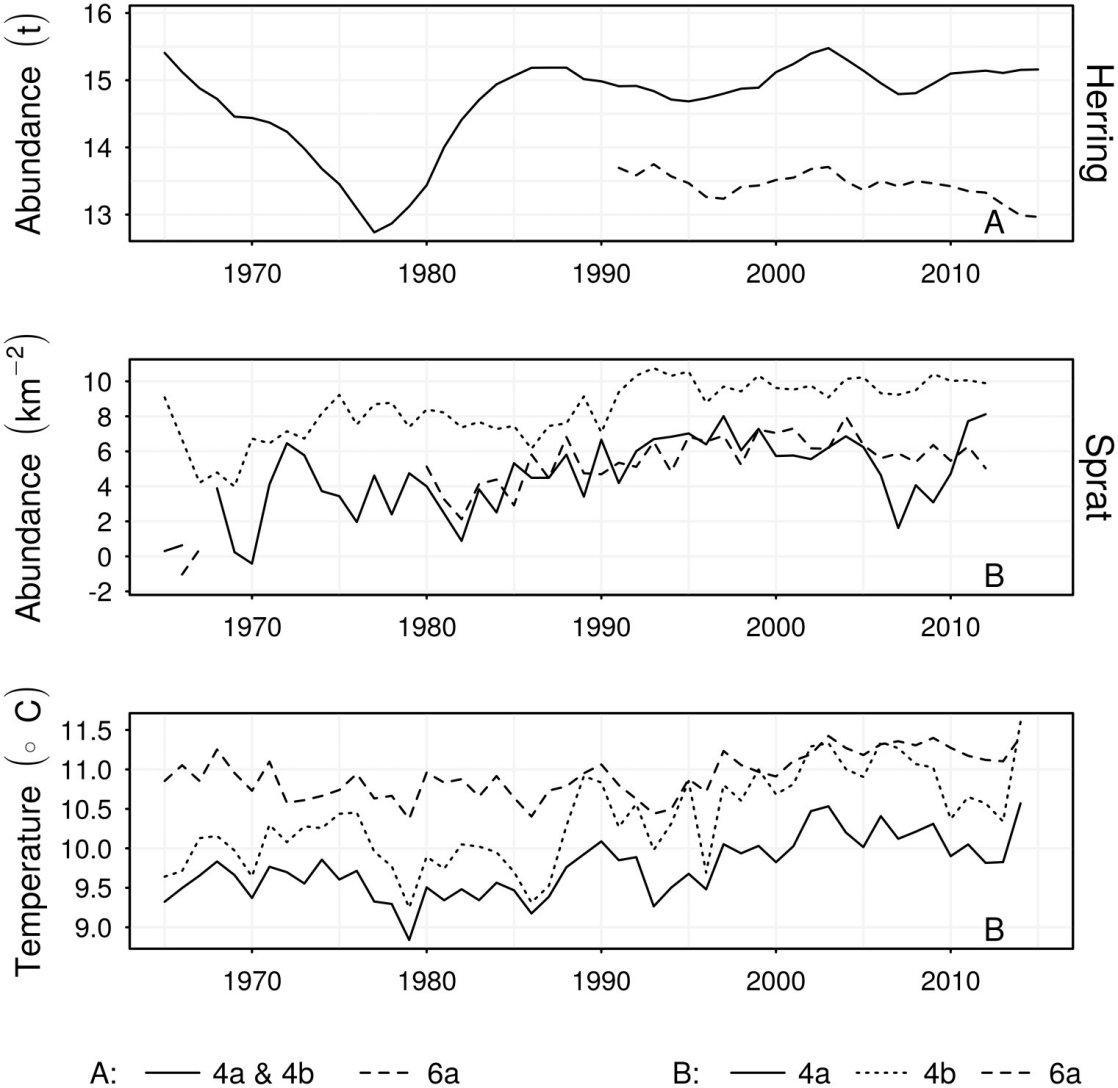
Time series of abundance estimates and temperature measurements. Each abundance time series is log_*e*_ transformed. Herring abundances are total stock biomass (tonnes) estimates from stock assessments. Sprat abundances are sampled numbers per km^2^ of area swept out by the survey trawl gear. Text in the bottom right corners refer to legends indicating region.

Annual mean summer sea-surface temperatures were calculated using the Hadley Centre HadISST1 data set [48]. In each region, mean monthly temperature was calculated as the mean over all measurements taken that month within that region; then summer temperature was calculated as the mean of mean monthly (June, July, and August) temperatures. Temperature during summer was used as a predictor of growth rates and maturation indices because this is when the majority of growth occurs, so temperature during summer is likely to be more influential upon growth than at any other time. Trends in abundance and temperature are shown in Fig 2.

### Age-length distributions

The quarter 1 age data were used to derive annual age-length keys specifying the distribution of ages within each length class. We used a continuation ratio method to generate smooth age-length keys [49] (details of this method are given in S2 Appendix). These were used to assign an age group to each fish in the length data, allowing estimation of total sampled numbers at age and length, hereafter referred to as the age-length data.

### Von Bertalanffy growth parameters

We calculated time series of mean lengths at age, and von Bertalanffy growth parameters using the age-length data. The growth parameters were estimated separately for individual cohorts using a maximum likelihood method [50] (see S3 Appendix for details).

As the set of three von Bertalanffy growth parameters are often highly correlated [51], considering trends in any one of the parameters in isolation can be misleading. Problems may arise when the data better represent the age-length structure of some cohorts over others. For example, when old and large individuals are not sampled from a cohort then the data cannot reliably inform the asymptotic length parameter, which may result in unrealistic *L*_∞_ values for that cohort. However, such unrealistic values tend to be compensated for by the other parameters, resulting in sensible predictions of length for the age groups present in the data. Although each growth parameter has its own physical interpretation, when analysing trends it is prudent to consider them jointly to avoid confounding correlations. We reduced potential for misinterpreting changes to individual growth parameters by analysing time series of absolute growth rates at age, given as the derivative of the von Bertalanffy length-at-age equation

$$\begin{array}{c} \Psi_{c}\left(a\right)=\frac{dl}{da}=k_{c}\left(L_{\infty \;c}-L_{0\;c}\right)\exp\left(-k_{c}\;a\right) \end{array}$$ (1)

where *l*, *a*, and *c* denote length, age, and cohort.

### Probabilistic maturation reaction norms

Trends in maturation were assessed using PMRNs derived for each cohort sampled for maturity status. A PMRN specifies the age and length dependent probability of first maturation; of transitioning from juvenile to adult. Information on first maturation was not present in the IBTS maturity data. We estimated the PMRNs following [52], because this method does not require data on first maturation, only data specifying the age and length of fish, and whether they are juvenile or mature.

We used PMRN midpoints, *L*_*p*50_, defined for each age group as the length corresponding to a 50% probability of first maturation, to assess trends in maturation. Within-age-group lengths at maturation were evaluated using time series estimates of PMRN midpoints, *L*_*p*50*c*_(*a*), for each cohort and age group with sufficient data. Increases in *L*_*p*50*c*_(*a*) for any given age group, indicate that lengths at first maturation have increased, thus correspond to a decline in maturation probability at length. Similarly, decreases in *L*_*p*50*c*_(*a*) correspond to an increase in maturation probability at length. PMRNs are largely independent of growth rates and mortality regimes, so changes in these factors should not influence estimates of trends in maturation [26].

Confidence intervals for *L*_*p*50_ estimates were generated by bootstrapping the maturity data. The data were resampled 1000 times with replacement, stratifying by cohort, to generate distributions for *L*_*p*50_. Confidence intervals were set at the 95% percentiles of the bootstrapped distributions. Mathematical details of the PMRN calculations are given in S4 Appendix.

### Temporal trends and the influence of abundance and temperature

GAMs were used to determine if trends in Ψ or *L*_*p*50_ were related to changing abundance or temperature. The time series of Ψ_*c*_(*a*) and *L*_*p*50*c*_(*a*) were each modelled as

$$\begin{array}{c} P_{c}=s\left(Y_{c}\right)+s\left(T_{c}\right)+s\left(\ln\left(N_{c}\right)\right) \end{array}$$ (2)

where *P*_*c*_ denotes either Ψ_*c*_(*a*) or *L*_*p*50*c*_(*a*), and the covariates are smoothing spline functions of temperature *T*_*c*_, abundance *N*_*c*_, and time *Y*_*c*_. Eq (2) was fitted separately to growth rates Ψ_*c*_(*a*) of age groups 1–5, and to *L*_*p*50*c*_(*a*) maturation indices of age groups 1–3. As these metrics were estimated for each cohort *c*, and the abundance and temperature measures related to year, the *N*_*c*_ and *T*_*c*_ time series were shifted *a* years to match the age group being modelled. As the history of stock abundance and temperature experienced by an individual may influence maturation probability, the *N*_*c*_ and *T*_*c*_ variables were calculated as the mean abundance or temperature over cohort *c*’s first *a* years of life when *P*_*c*_ = *L*_*p*50*c*_(*a*). This was not done when *P*_*c*_ = Ψ_*c*_(*a*) because Ψ_*c*_(*a*) is the instantaneous growth rate. These models were fitted using the *gamm* function from the *mgcv* R package. Each model was fitted assuming Gaussian noise and with the identity link function. Potential for auto-correlation in the error structure was accounted for by mixing auto-regressive models within Eq (2) using options provided in the *gamm* function. Four models were fitted in each case: the base model, Eq (2); and three models including auto-regressive terms, AR1–AR3. From these four, we selected the best model based on AIC score, which was usually the base Eq (2) or AR1 model.

## Results

### Trends in length at age

**Fig 3:**
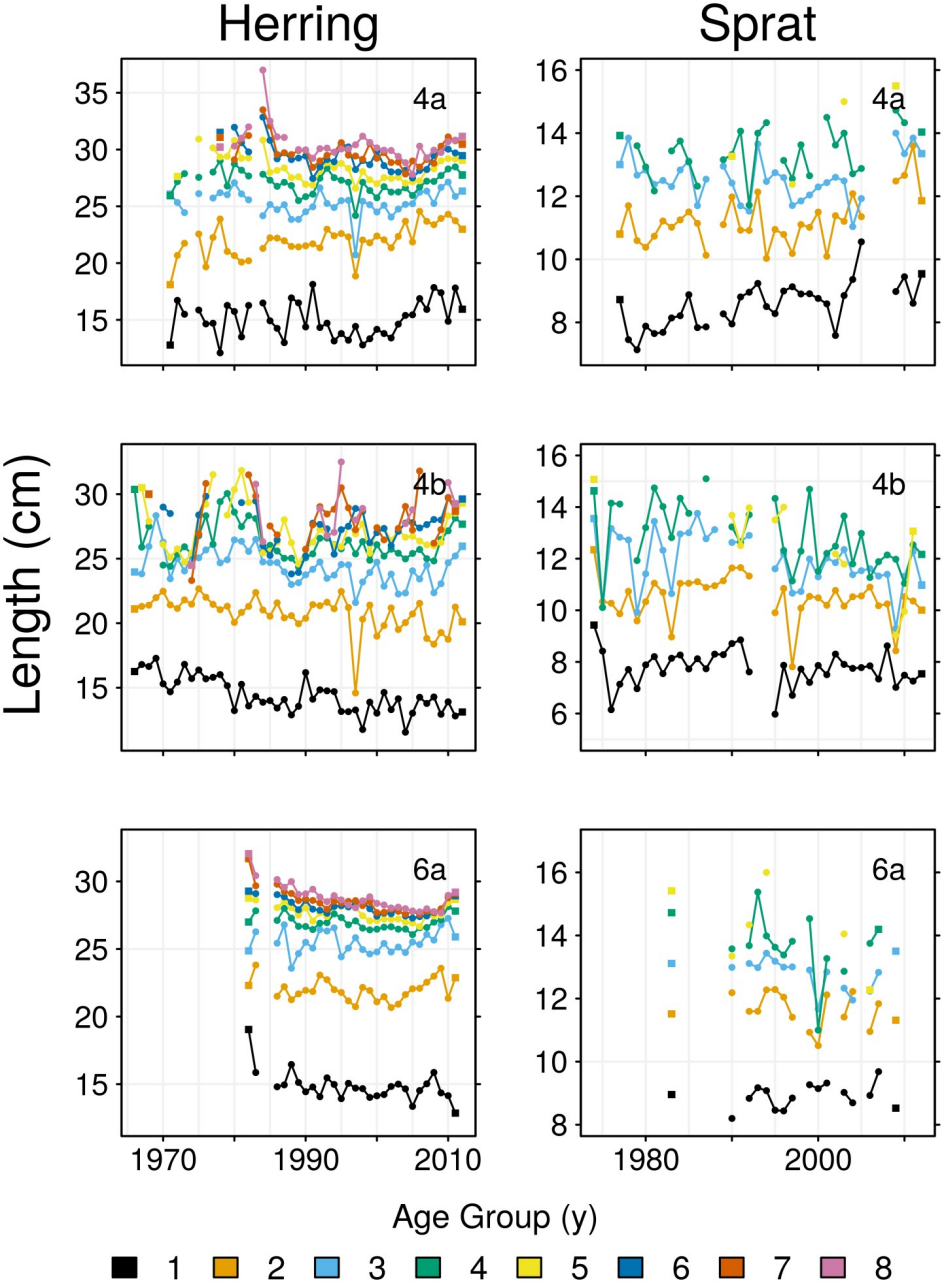
Trends in mean length at age. Square symbols indicate the initial and final years in the time series for each age group. Text at the top right corner indicates region.

The mean lengths of 4a herring in age groups 4–8 declined by 1–3 cm during 1980–2000, then partially recovered in the 2000s (Fig 3). Age group 2 gradually increased 2 cm in length during 1970–2010. The mean lengths of 4a sprat in age groups 1–4 increased by 0.5–1.5 cm during 1977–2012: the 2.5 cm increase in the length of age group 5 should be treated with caution as it is based on only four data points. Area 4b was typified by declines in length at age. The mean lengths of herring in age groups 1 and 2 declined by 1.5–2.5 cm during 1966–2012. Although the mean length of older age groups also decreased during much of this time, long-term trends were less pronounced as their lengths tended to increase during the late 2000s. Sprat in age groups 3–5 declined in mean length by 1–2 cm during 1974–2012 in area 4b. Declines in length at age were also evident in area 6a, where the mean lengths of herring in age groups 4–8 decreased by 1–2 cm between 1980 and 2005, before partially recovering during the late 2000s. Mean lengths of 6a sprat in age groups 3–5 declined by 0.5–2 cm.

### Growth parameters and absolute growth rate

**Fig 4:**
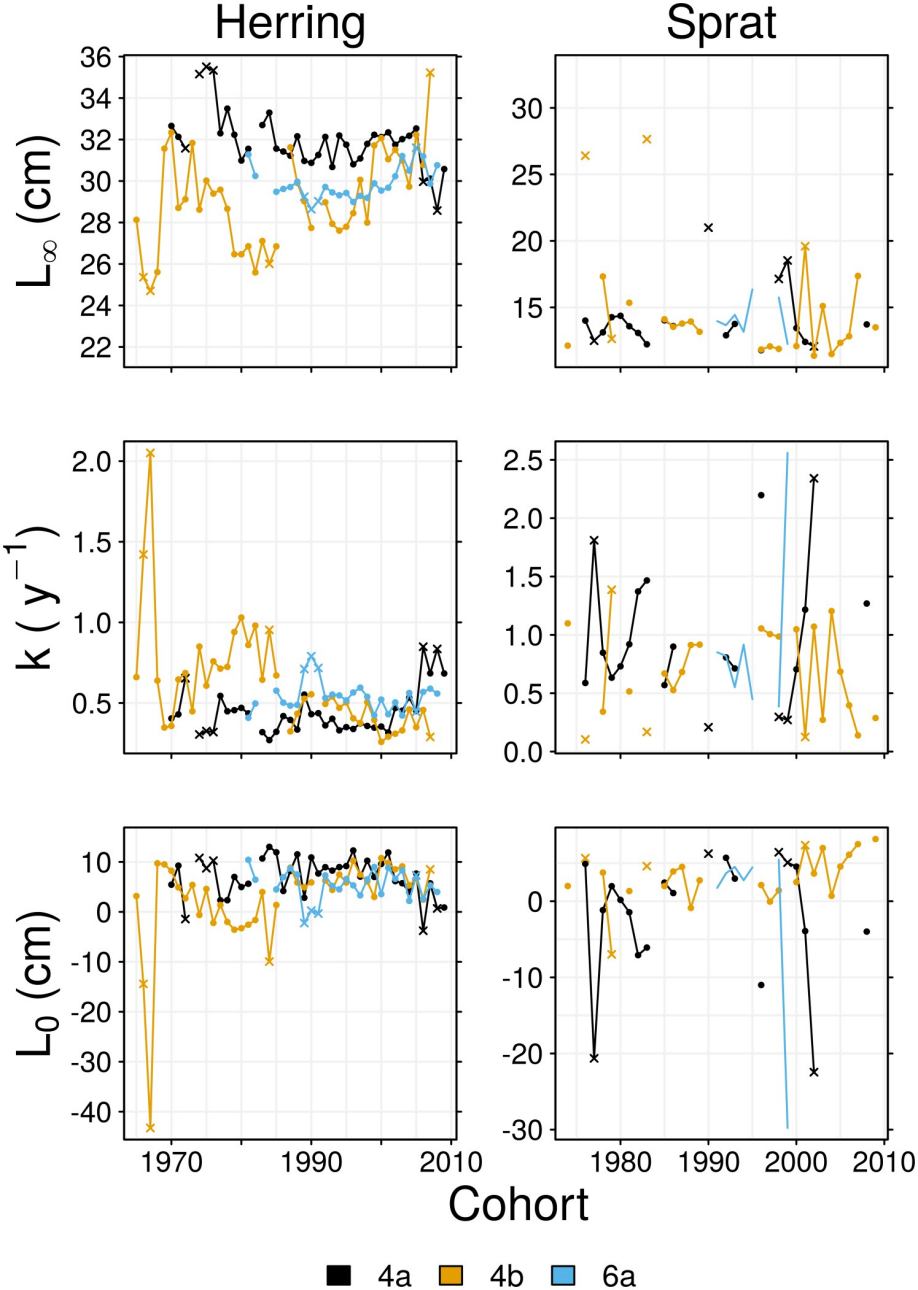
Time series of von Bertalanffy growth parameters. As these were separately estimated for each year group, the abscissa represents cohort, not year. Crosses indicate cohorts where at least one of the parameters is an outlying value.

**Fig 5:**
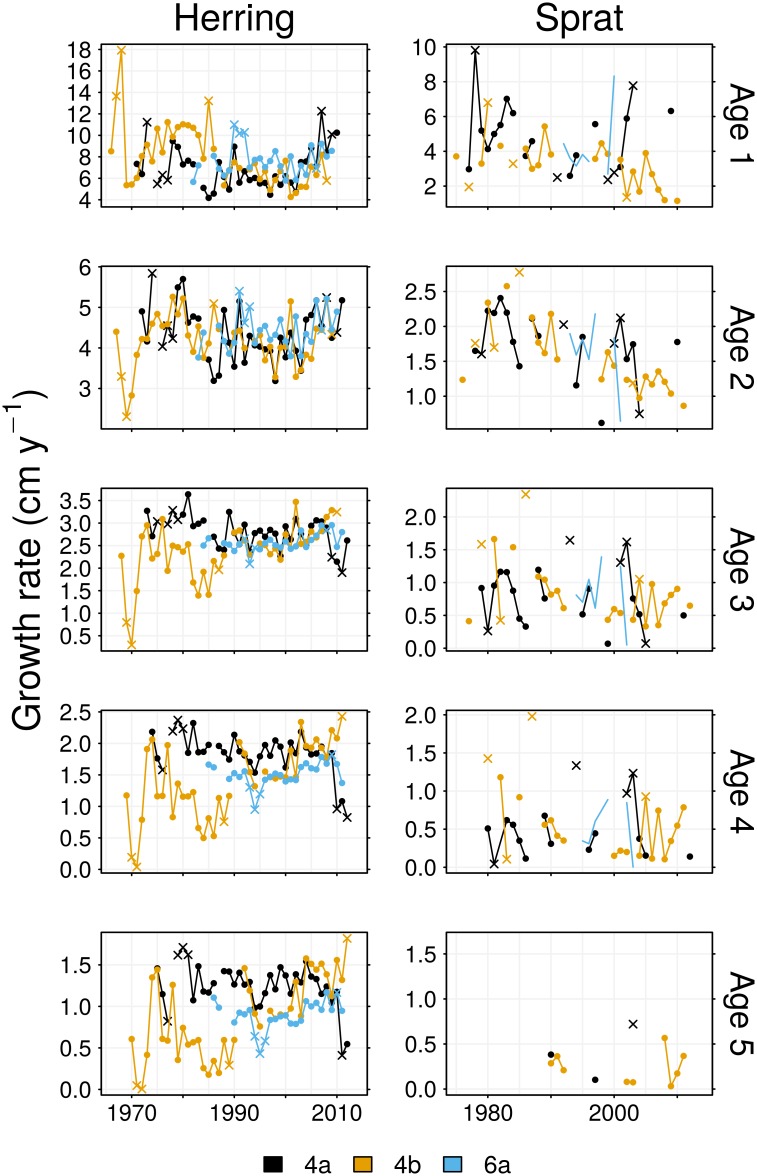
Growth rates at age, Ψ(*a*), calculated from Eq (1). Only age groups present in the data are represented in the plot. Crosses indicate cohorts where at least one of the growth parameters is an outlying value.

The asymptotic length, *L*_∞_, of 4a herring declined over time, but this was compensated by an increase in von Bertalanffy growth rate, *k*, during the late 2000s (Fig 4). This resulted in reduced variability in the mean lengths of age groups 3–8 by the late 2000s, as the population tended to grow more quickly to a smaller maximum size. Different trends were observed for 4b herring, as *L*_∞_ tended to increase over time while *k* tended to decrease. This represented declining growth rates of age groups 1 and 2 and, by the later half of the time series, the increased growth rates of older fish (Fig 5). The outlying parameter estimates of the 1966 and 1967 cohorts (Fig 4) resulted from these samples lacking old and large individuals; the mean lengths of relatively old age groups sampled from these cohorts were less than the mean length of age group 3, hence estimated growth rates approach zero from age group 3 because this is when these cohorts approach full size (Fig 5). Changes to the growth of 6a herring were most evident in old age groups, and were characterised by a declining *L*_∞_ during 1980–1995, followed by an increase in *L*_∞_ in the 2000s.

High variability in the growth parameter estimates for sprat made trends difficult to interpret (Fig 4), but the resulting growth rates at age were more stable (Fig 5). The observed increases in the mean lengths of 4a sprat appear to be largely due to an increase in initial length *L*_0_, as growth rates at age show no sign of an upward trend. Declines in the lengths of 4b sprat were mainly due to decreases in the growth rates of age groups 1 and 2. Trends in the growth parameters of 6a sprat are not obvious as there are too few estimates, although there is a suggestion of a decrease in growth rate in the mid 1990s in age groups 2 and older, which would explain the observed decline in mean lengths.

### Trends in maturity at age and *L*_*p*50_

**Fig 6:**
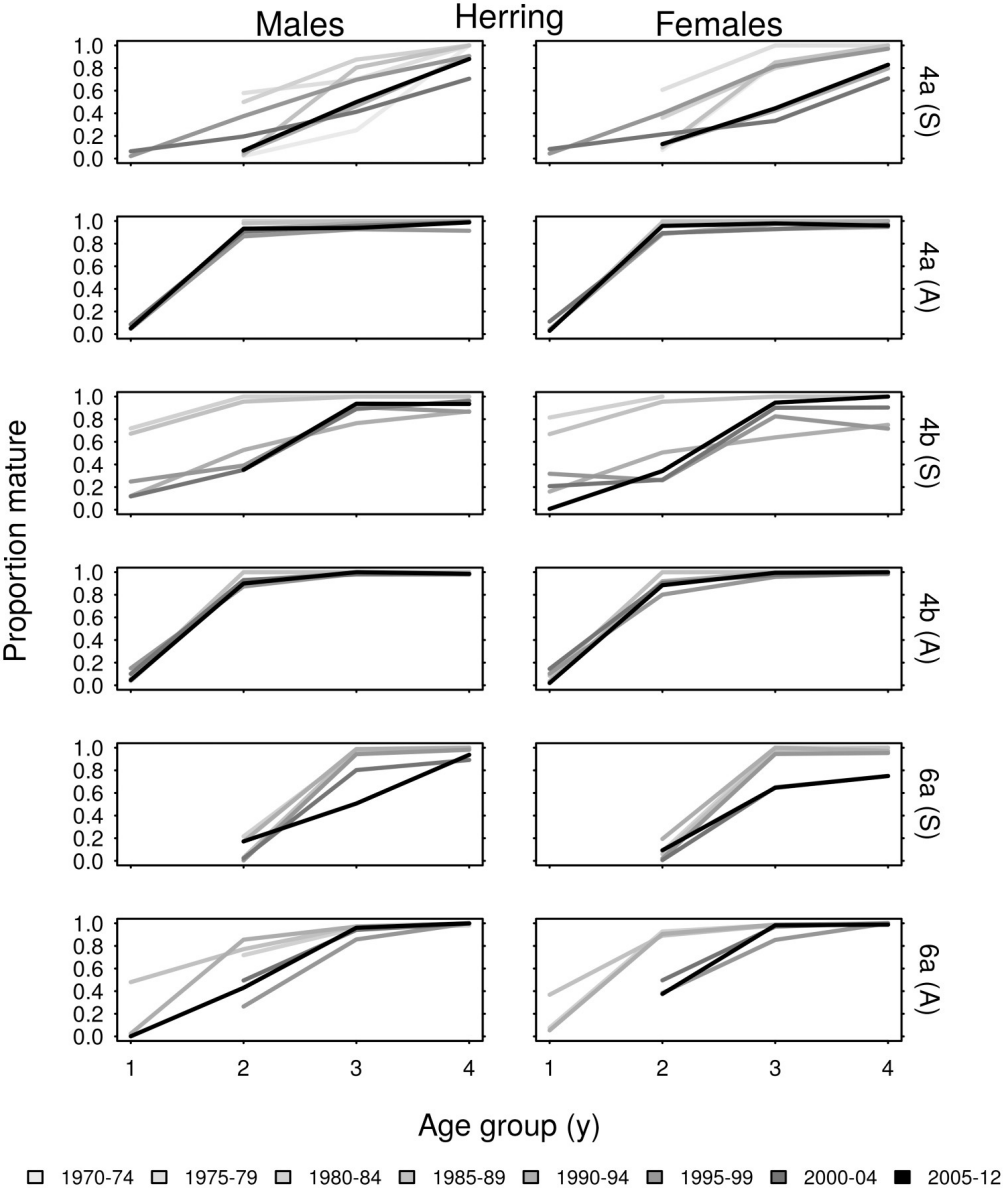
The proportion of sampled age group 1–4 herring that were mature, averaged over five year intervals. Spring and autumn spawning herring are indicated by (S) and (A) respectively.

**Fig 7:**
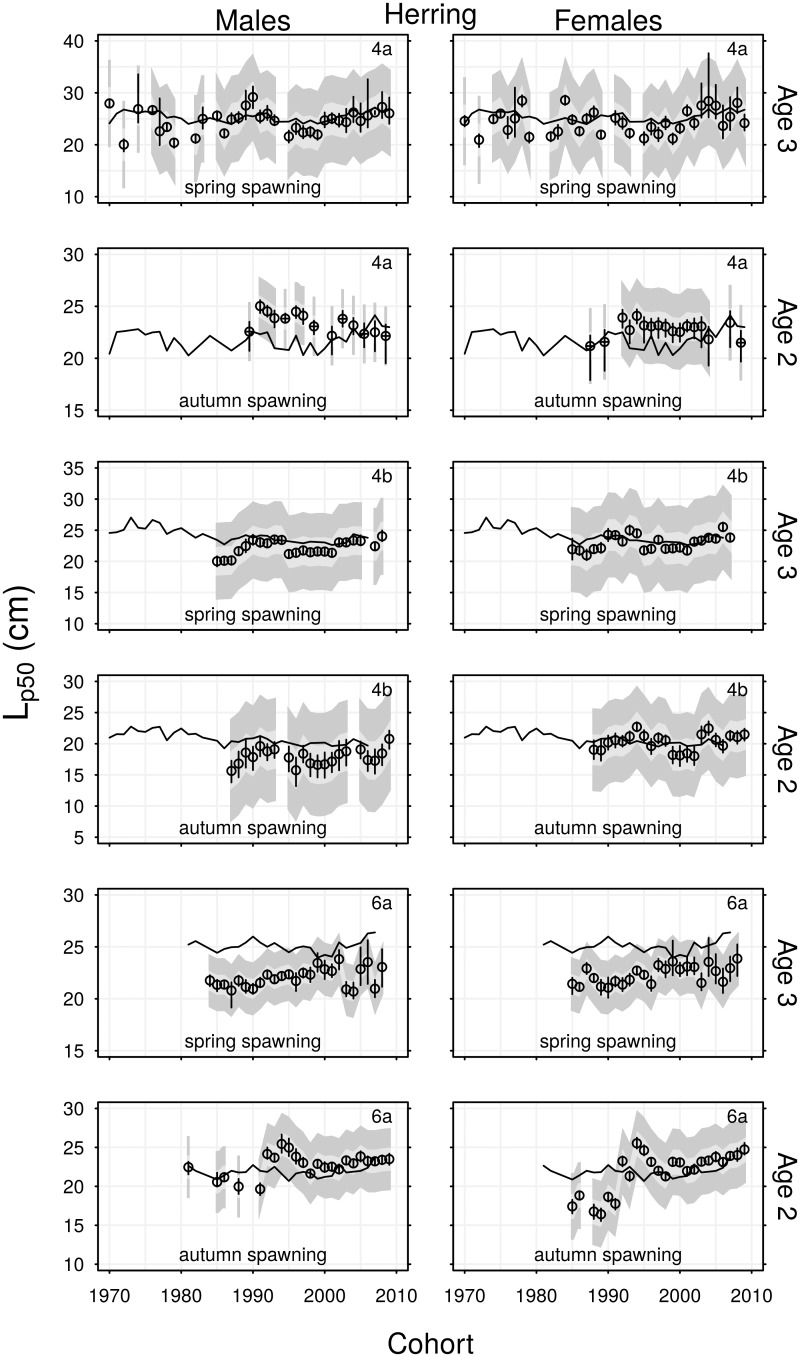
Time series of *L*_*p*50_ estimates for cohorts of herring. Vertical lines are 95% confidence intervals. Light and dark grey polygons are maturation envelopes, representing *L*_*p*25_ → *L*_*p*75_ and *L*_*p*05_ → *L*_*p*95_ respectively. Horizontal lines show expected length at age based on the von Bertalanffy growth parameters. Text in the top-right corner indicates region.

There was little change over time in the proportion of North Sea autumn spawning herring that were mature within each age group, and the majority of these fish were mature by age 2 (Fig 6). The *L*_*p*50_ values of males and females in age group 2 were in the range 21–25 cm in area 4a, and 15–23 cm in area 4b (Fig 7). Figures display *L*_*p*50_(*a*) values for the age groups in which most fish matured; there was little difference in *L*_*p*50_ trends between age groups. Although the North Sea autumn spawning herring *L*_*p*50_ values varied over time, the only consistent long-term trend was a decline in *L*_*p*50_ of males from area 4a.

The proportion of North Sea spring spawning herring that were mature within age each group varied more substantially and tended to decrease over time. The majority of these fish were mature by age 3. The *L*_*p*50_ values of spring spawners vary in the range 20–29cm in area 4a, and 20–25 cm in area 4b, with a general tendency to increase over time. The initial decrease in proportions mature at age in area 4b, can be clearly seen as an increase to *L*_*p*50_ during 1985–1995.

The proportions of spring and autumn spawning herring from area 6a that were mature within each age group declined over time. This was evident in the estimated *L*_*p*50_ values, which tended to increase over time, representing reduced probabilities of maturation. Long-term trends in *L*_*p*50_ were more evident in 6a herring than those from the North Sea.

**Fig 8:**
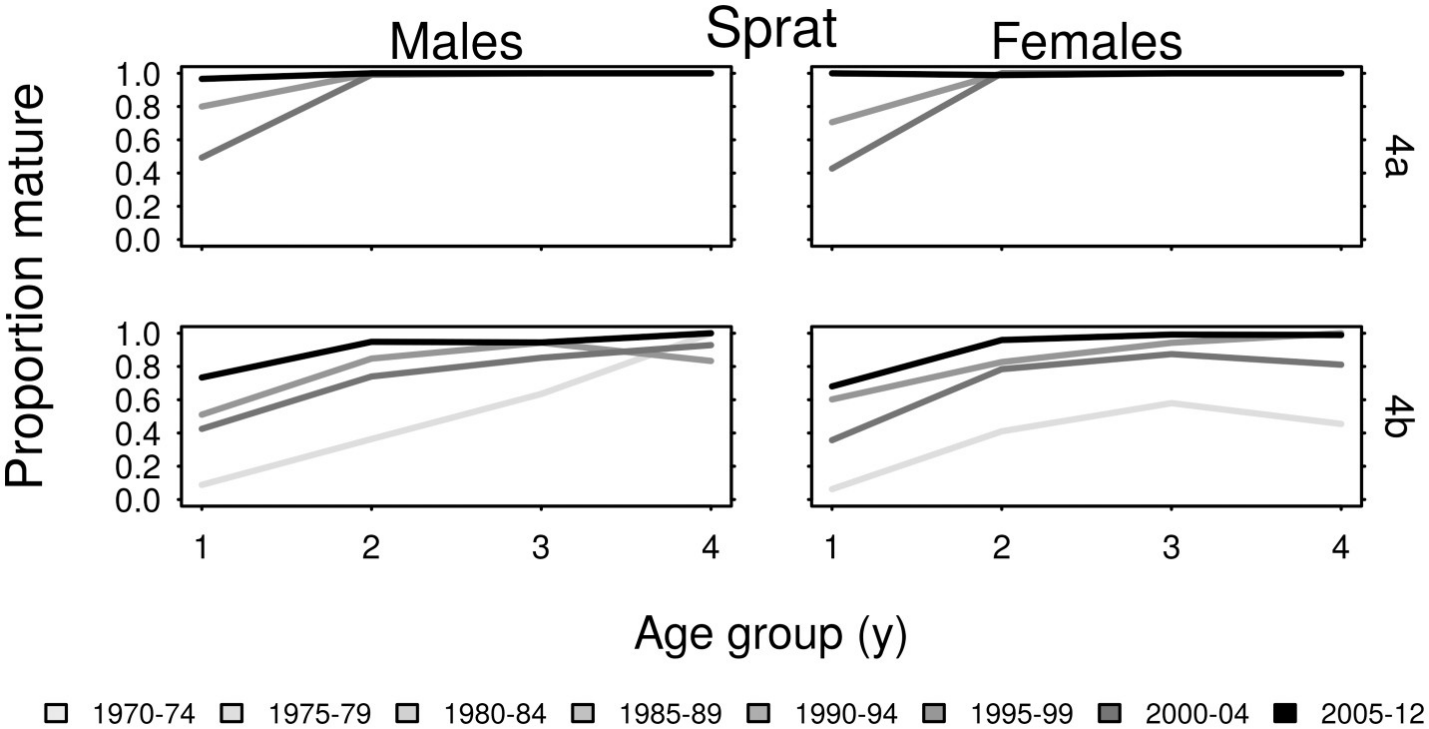
The proportion of sampled age group 1–4 sprat that were mature, averaged over five year intervals.

**Fig 9:**
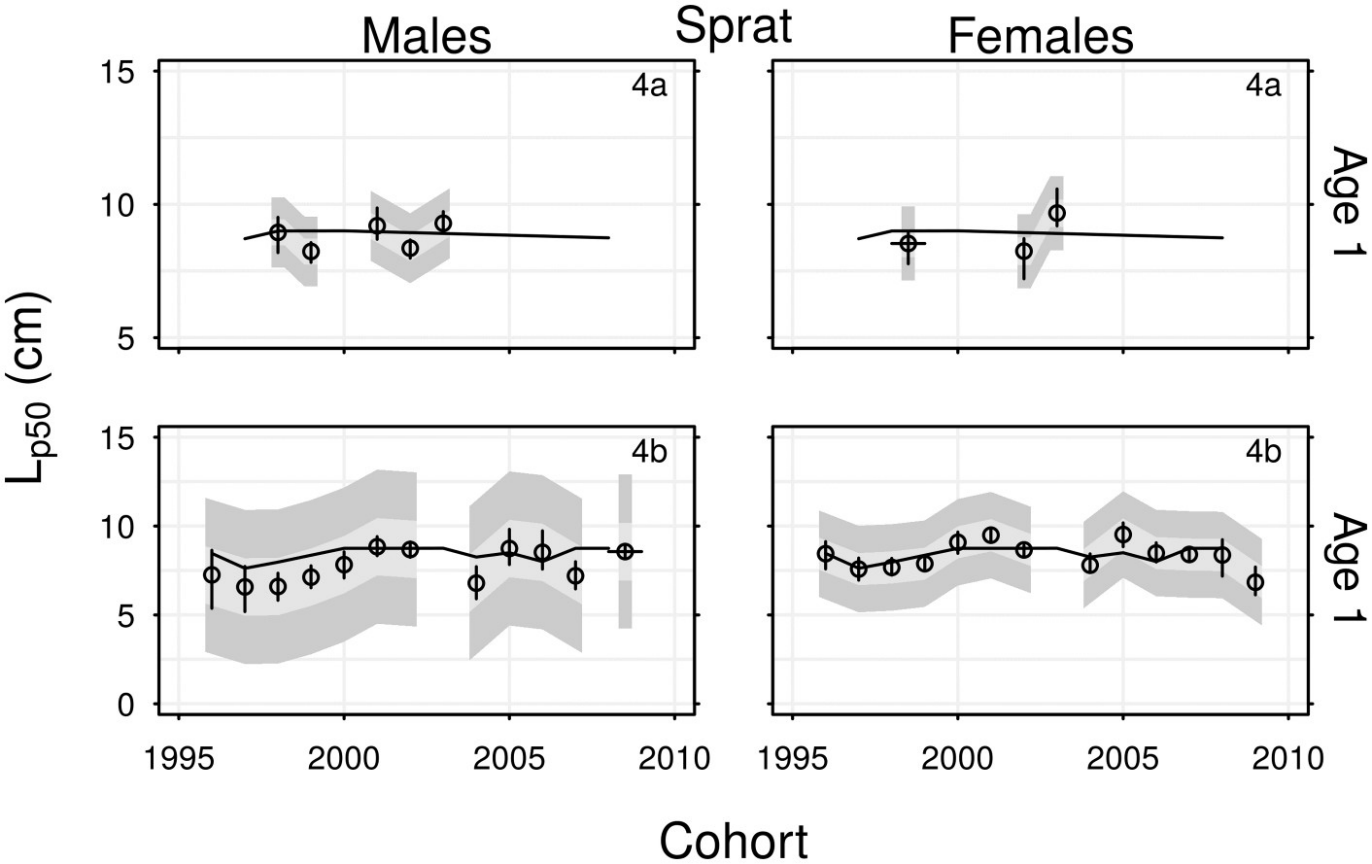
Time series of *L*_*p*50_ estimates for cohorts of sprat. Vertical lines are 95% confidence intervals. Light and dark grey polygons are maturation envelopes, representing *L*_*p*25_ → *L*_*p*75_ and *L*_*p*05_ → *L*_*p*95_ respectively. Horizontal lines show expected length at age based on the von Bertalanffy growth parameters. Text in the top-right corner indicates region.

The proportion of North Sea sprat that were mature within each age group tended to increase over time (Fig 8). Sprat in area 4a were mature by age 2, and most were mature at age 1. There were higher proportions of immature sprat within age groups in area 4b. As limited maturity data for 4a sprat meant that *L*_*p*50_ could only be estimated for three female cohorts and five male cohorts it was not possible to detect trends, but the *L*_*p*50_ values were in the range 8–9.5 cm (Fig 9). The *L*_*p*50_ values of the 1996–2009 cohorts of 4b sprat fluctuated within the range 6.5–9.5 cm, without exhibiting consistent long-term trends.

Expected lengths at age are included in Figs 7 and 9 for comparison with *L*_*p*50_ estimates. Expected lengths greater than *L*_*p*50_ indicate that the majority of individuals from an age group have maturation probability greater than 50% (e.g. 6a spring spawning herring in age group 3). Expected lengths less than *L*_*p*50_ indicate that only relatively large individuals from an age group have 50% maturation probability. This is most clearly observed in 4a autumn spawning herring in age group 2, but note that as expected lengths were calculated using spring data, the majority of these age group 2 fish will mature by autumn since their lengths increase over summer.

### The influence of abundance and temperature

The GAM analyses show that growth rates, Ψ, of North Sea herring and sprat were negatively correlated with abundance (Table 2). These negative correlations were evident in herring of age groups 2–5, and sprat in age groups 1–3. Growth rates in area 6a were not related to abundances. The effect of temperature upon growth rates differed between species. Growth rates of 6a herring in age groups 1, 3 and 4 were positively correlated to temperature, as was the growth rate of age group 4 in area 4a. The growth rates 4b sprat in age groups 3 and 4 were negatively correlated with temperature; negative and non-linear relationships between growth rate and temperature were also evident in 4a sprat in these age groups. Although abundance and temperature has been shown to correlate significantly with trends in growth rates, some variability in growth rates remains unaccounted for, as shown by the significant cohort effects representing temporal trends (Table 2).

**Table 2:** Summary of results from GAM analyses of growth rates. The Effect column groups model results by covariate type. Columns 1–5 represent the models for growth rate Ψ in age groups 1–5. Positive and negative correlations between Ψ and the covariates are represented by orange and blue cells respectively. Yellow cells represent significant non-linear relationships. Groups without data are coloured grey. Statistical significance was approximated using *p*-values: *** *p* < 0.001; ** *p* < 0.01; * *p* < 0.05;. *p* < 0.1.

Effect	Species	Region	1	2	3	4	5
cohort	herring	4a	**		*	***	***
cohort	herring	4b	.	**	***	***	***
cohort	herring	6a	***	**			*
cohort	sprat	4a				*	
cohort	sprat	4b	*		*		
cohort	sprat	6a		.			
temperature	herring	4a				**	
temperature	herring	4b					
temperature	herring	6a	***		**	*	
temperature	sprat	4a			.	*	
temperature	sprat	4b			**	**	
temperature	sprat	6a					
abundance	herring	4a		**		***	**
abundance	herring	4b		***	**	**	**
abundance	herring	6a					
abundance	sprat	4a	.	.			
abundance	sprat	4b		*	**		
abundance	sprat	6a					

The abundance of autumn spawning herring is negatively correlated with *L*_*p*50_ in most instances; the exception being 4b males with no significant effect (Table 3). The only other significant abundance effect is a negative correlation with *L*_*p*50_ of 4b spring spawning females: the abundance of sprat did not influence *L*_*p*50_. As observed in the GAM analyses of growth, the effect of temperature upon *L*_*p*50_ differed between species. The *L*_*p*50_ values of North Sea autumn spawning male herring were negatively correlated with temperature. A negative temperature correlation was also observed in spring spawning males in area 4b, and some non-linear temperature effect in 6a spring spawning females. Whereas the *L*_*p*50_ values of 4b sprat males and females were positively correlated with temperature. Although abundance and temperature correlate with the *L*_*p*50_ time series, some positive and non-linear changes over time remain unaccounted for, as shown by the significant cohort effects in Table 3.

**Table 3:** Summary of results from GAM analyses of *L*_*p*50_. The Effect column groups model results by covariate type. Columns 4a, 4b, and 6a represent the GAM models for *L*_*p*50_ in each region. Autumn and spring spawning herring are distinguished by (A) and (S). Positive and negative correlations between Ψ and the covariates are represented by orange and blue cells respectively. Yellow cells represent significant non-linear relationships. Groups without data are coloured grey. Statistical significance was approximated using *p*-values: *** *p* < 0.001; ** *p* < 0.01; * *p* < 0.05;. *p* < 0.1.

Effect	Species	Sex	Age	4a	4b	6a
cohort	herring (A)	F	2	*	**	.
cohort	herring (A)	M	2	***	*	
cohort	herring (S)	F	3		**	*
cohort	herring (S)	M	3		***	
cohort	sprat	F	1			
cohort	sprat	M	1		.	
temperature	herring (A)	F	2			
temperature	herring (A)	M	2	**	*	
temperature	herring (S)	F	3			.
temperature	herring (S)	M	3		.	
temperature	sprat	F	1		**	
temperature	sprat	M	1		*	
abundance	herring (A)	F	2	**	***	***
abundance	herring (A)	M	2	***		**
abundance	herring (S)	F	3		**	
abundance	herring (S)	M	3			
abundance	sprat	F	1			
abundance	sprat	M	1			

## Discussion

We found negative correlations between growth rates at age, Ψ(*a*), and abundance for herring and sprat (Table 2). In agreement with density-dependent growth theory [53], the growth rates of North Sea herring and sprat tended to decrease in response to increases in abundance. This result agrees with studies of Baltic Sea sprat which found body condition (used as a growth index) to decrease with rises in abundance [23, 54]. Particularly evident was the effect of the decline and subsequent increase in North Sea herring abundance in the 1970s and 1980s upon growth rates (Figs 1 and 5). Growth rates were relatively high during the low abundance period of the 1970s and the early 1980s, then declined when the herring stock recovered. This result corresponds to the timing of the largest change in the North Sea herring length anomaly reported in [55]. The growth rates of 4b herring then tended to increase from the 1990s (Fig 5 and Table 2). Rising sea surface temperatures were correlated with slowed growth rates of 4b sprat in age groups 2–4. In contrast, rising temperatures were positively correlated to herring growth rates, particularly in area 6a, and to lesser extent area 4a. It has been speculated that temperature variation in the North Sea could potentially explain inverse patterns of recruitment success of herring and sprat [55]. While we have not examined recruitment success, our results tie in to this idea by demonstrating an inverse pattern of growth rate responses to temperature. Metabolic rates and oxygen requirements increase with rising temperature, while dissolved oxygen concentrations decrease [56]. Thus, growth rates may increase with temperature provided fish can satisfy metabolic demands, but growth rates will inevitably decline once temperature exceeds the threshold where circulatory and ventilatory systems are strained to meet oxygen requirements [57]. We have found mixed growth responses to rising temperature: perhaps the slowed growth of 4b sprat is due to increased metabolic costs driven by temperature increases. We have shown that abundance and temperature fluctuations have influenced growth rates. Increases in stock abundances have, in general, tended to suppress growth rates, reducing lengths at age. However, as the time covariate was significant for many of the GAMs of Ψ, it appears that growth rates have also been responding to factors not considered in this study; this is discussed further below. It should be noted that the significant non-linear cohort effects on growth rates in 6a herring are most likely an artifact of stock assessment abundance metrics not covering the full time series of growth rate estimates (Figs 2 and 4).

Negative correlations between *L*_*p*50_ and autumn spawning herring abundance were present in all three regions. This indicates that maturation is likely to occur earlier, at smaller sizes, when abundance is high. In general, increases in abundance of North Sea herring appear to have suppressed *L*_*p*50_ values, particularly in area 4a, whereas the decline in herring abundance in area 6a (Fig 2) may have raised *L*_*p*50_ in this region. These results align with density dependent effects reported in Norwegian spring spawning herring [22]. Correlation between abundance and *L*_*p*50_ was less evident for spring spawning herring in our study, although some negative correlation was observed for females in area 4b. In general, rising temperature has increased the age-length dependent maturation probabilities of male herring, while decreasing maturation probabilities of sprat. This was demonstrated by negative and positive correlations between temperature and *L*_*p*50_ for herring and sprat respectively. The different temperature regimes of the northern and southern North Sea appear to have similarly influenced maturation schedules. What was much more apparent in this study were the consistent differences in species response, in terms of both maturation and growth. Clearly the age and length dependent maturation schedules of herring and sprat in these regions have some consistent responses to fluctuations in abundance and temperature. However, several significantly positive and non-linear responses over time also indicate maturation responses to other factors.

Growth rates and maturation schedules may respond to a variety of environmental changes other than abundance and temperature. Food availability is of particular importance. The amount of food available per individual is the main determinant of growth rate [58], and *L*_∞_ is directly related to food intake [25]. Trends in the abundance of food can therefore cause changes to average lengths at age and maximum lengths. The response of herring and sprat growth to zooplankton abundance was studied by [55], who found that the abundance of a common zooplankton species was positively and negatively correlated to the growth of herring and sprat respectively. As different stocks are exposed to different prey, have different prey preferences, and possibly respond differently to changes in food availability [55], accounting for the influence of food availability upon growth and maturation is fraught with difficulty. Changes in length at age should not influence PMRN positions [26], however, food availability may affect *L*_*p*50_ values by altering the average condition (weight at length) of fish populations, as individuals in poor condition are more likely to delay maturation [59]. We did not consider trends in food availability for reasons of data availability and interpretation, but as doing so may lead to further insight on some of the unexplained temporal trends in growth rates and *L*_*p*50_, such work could usefully extend this study.

Species with overlapping distributions and diets may compete for resources, so inter-specific competition may have influenced the growth of herring and sprat. Sprat are planktivorous, feeding on copepods throughout their entire lives [60]. Herring are omnivorous, they feed exclusively on zooplankton until they grow large enough to handle larger prey items, at which point they may also consume small fish including sprat [60]. We might therefore expect these species to compete for zooplankton. Fluctuations in the abundance of either species may then influence growth of the other through inter-specific competition. Mackerel (*Scomber scombrus*) tend to be larger than herring, and they are faster swimmers, more efficient plankton eaters, and usually have fuller stomachs [61], so are potentially the main competitor to herring within the northern North Sea. As mackerel are less abundant in the southern North Sea, sprat are perhaps a more significant competitor to herring as they are highly abundant in this region. It may be useful for further studies to consider how changes in the abundance of competing species in the North Sea and west of Scotland potentially influence growth (e.g. [62, 63]). This would be challenging, however, as it would involve determining which species are in direct competition for certain food resources, and should account for each species prey preferences and omnivory.

Relaxation of predation pressures due to overfishing may induce changes in the growth of prey species [11]. Declines in the abundance and size of piscivorous demersal species may have reduced the mortality rates of the pelagic species considered here, particularly the mortality rates of young/small individuals. This could lead to increased abundance, greater competition, and consequent reductions in growth. Increased abundance of juveniles due to suppressed predation rates may also lead to declines in condition and delayed maturation [59]. In addition to reducing mortality rates, declines in predation can induce changes in the foraging behaviour of prey species such as herring and sprat [64], which may also influence growth rates. Predators can only consume prey that is within certain size-ranges [65], so fishing- and climate-induced declines in the size of predatory fish [17, 66] may have reduced the size at which prey species become invulnerable to the majority of predators, thus relaxing selective pressures to grow and mature quickly. Of course it is not only other fish species that predate herring and sprat; changes in the predation of marine mammals and seabirds may also be influential.

Fishing is size-selective, imposing a disproportionately high mortality rate upon large individuals, so fishing activity may have contributed directly to changes in the growth and maturation of the North Sea and west of Scotland herring and sprat. As fishing lowers the abundance of harvested populations it may, through reduced competition, increase food-availability and average growth rates [53]. Fishing gears predominantly harvest large individuals, allowing smaller fish to escape, and therefore impose selective pressures that are opposite to those of predation [67]. This may lead to suppressed growth rates and early maturation through evolutionary processes, especially when minimum landing sizes are close to typical sizes at maturation [3]. Since the pelagic fish considered here are small compared to most demersal fish species thought to be subject to fisheries-induced evolution, fishing is perhaps more likely to affect growth and maturation of herring and sprat indirectly through altering food availability and the size-structures and abundances of predator populations. Trends in the growth and maturation of North Sea and west of Scotland herring and sprat may be partially caused by trends in the abundance of competitor and predator species, and trends in the average size of predators; further investigation into these potential relationships would be complex but may add more detail to our understanding of trends in growth and maturation.

## Supporting information

S1 Appendix Categorising herring as spring or autumn spawners. (PDF)

S2 Appendix Age-length distributions. (PDF)

S3 Appendix Von Bertalanffy growth parameters. (PDF)

S4 Appendix Probabilistic maturation reaction norms. (PDF)
