# Supplementary material for: Population density and temperature correlate with long-term trends in somatic growth rates and maturation schedules of herring and sprat
Source: PLoS One. 2019 Mar 6;14(3):e0212176. doi: 10.1371/journal.pone.0212176 (PMC6402831; doi:10.1371/journal.pone.0212176)
Supplement: S1 Appendix — (PDF) [file pone.0212176.s001.pdf]

The maturation analyses were conducted separately for spring and autumn spawning herring populations. Each individual within the maturity data was categorised as a spring or autumn spawner. Spring and autumn spawning herring were not considered separately for the growth analyses because the categorisation process involved the von Bertalanffy growth parameters. The maturity status of sampled herring may be used to determine whether individuals were from spring spawning or autumn spawning populations, provided that the samples were collected in the period from two months prior to spawning season until the peak of the spawning season [1]. The method is thought to correctly identify the bulk of sampled herring as spring or autumn spawners, however, correctly distinguishing every individual is unlikely. We used survey data collected prior to spring and autumn spawning, in quarters 1 and 3. The eight-stage maturity scale commonly used for herring is: (1) virgin, (2) virgin with small gonads, (3–5) maturing, (6) spawning, (7) spent, and (8) recovering [2]. During quarter one, adult spring spawners will be maturing while autumn spawners will be spent or recovering, so fish in maturity stages 3–6 can be classed as spring spawners, while those in stages 7–8 or 2 are autumn spawners. It is the reverse during quarter three, when fish in maturity stages 3–6 can be classed as autumn spawners, and those in stages 7–8 or 2 are spring spawners [1]. If a survey uses an eight-stage maturity scale such as this, then the adult herring (stages 2–8) present in samples collected during quarters one or three may be categorised as spring or autumn spawners.

The IBTS data set has not used this eight-stage maturity scale. Instead maturity was classed as: (1) juvenile, (2) maturing, (3) spawning, and (4) spent. As above, spawning and spent individuals can be categorised as belonging to spring or autumn spawning populations. Stage 2 individuals, however, need to be split into two groups corresponding to stages 2 and 3–5 on the eight-stage maturity scale. The FRS data set consisted of a collection of different surveys which included the IBTS data, so the maturity status of an individual within the FRS data set may have been recorded using the eight-stage scale or the less informative IBTS scale. A ‘test’ data set that only used the eight-stage maturity scale was obtained by extracting FRS data collected in years where the IBTS surveys were not conducted. Every mature fish (stages 2–8) in this

'test' data set was then classed as spring or autumn spawning. The rest of this section describes how the remaining uncategorised herring were assigned to either spring spawning or autumn spawning populations. The remaining uncategorised herring consisted of the juveniles (stage 1 on either scale) and fish recorded as maturity stage 2 in the IBTS data.

The recorded age of herring had been standardised so that the assumed birthdays were the January 1st nearest to the time of spawning. Therefore, within age groups, autumn spawned individuals will be approximately six months older, and often larger on average, than spring spawned fish. Plots of length distributions within age groups were often bimodal or highly skewed, suggesting the presence of both autumn and spring spawned fish. Mixture-normal distributions were fitted to the annual length distributions for age groups 1–4 to assess whether the non-normality could be due to distributions of length at age differing between spring and autumn spawners. Expected within-age-group lengths were calculated from the time series of von Bertalanffy growth parameter estimates using approximate 'true' birthdays for spring and autumn spawned herring. These 'true' birthdays were January $1^{\text{st}} \pm 3$ months. The mixture-normal distributions and the expected lengths of spring and autumn spawners were then overlaid on plots of the length frequency distributions. The expected lengths of spring and autumn spawners were a close match to the two peaks of the mixture-normal distributions for many of the years where the distributions of length at age were bimodal or skewed. In these instances, each individual not previously categorised via recorded maturity statuses was classed as a spring or autumn spawner using the mixture-normal distributions. Thus, within-age-group length frequency distributions combined with predicted lengths at age were used to categorise many of the juvenile (stage 1) and IBTS stage 2 herring as spring or autumn spawners. The FRS 'test' data set was then expanded to include these newly categorised individuals.

The remaining juvenile and IBTS stage 2 herring were categorised as spring or autumn spawners by fitting GAMs to the 'test' data. The GAMs modelled the probability of being a spring or autumn spawner against smooth functions of age group, a, and length, l. Further covariates, included when significant ($p < 0.05$), were year, y,

area partitions by statistical rectangle, sr, and sex, s.

$$\text{logit}(p(\text{autumn}, \text{spring})) = \alpha_{sr} + \alpha_s + f(a, l) + y\, g(a, l) \tag{1}$$

The IBTS stage 2 fish were considered first, so the data used to fit Eq (1) consisted of all adults within the 'test' data. Eq (1) was then fitted to juveniles from the 'test' data. The fitted GAMs were then used to categorise the remaining herring as belonging to spring or autumn spawning populations.

References

1. Bucholtz RH, Tomkiewicz J, Dalskov J. Manual to Determine Gonadal Maturity of Herring (*Clupea harengus* L.). DTU Aqua-report 197-08. Charlottenlund: National Institute of Aquatic Resources; 2008.

2. McPherson LR, Ganias K, Marshall CT. Inaccuracies in routinely collected Atlantic herring (Clupea harengus) maturity data and correction using a gonadosomatic index model. Journal of the Marine Biological Association of the UK. 2011;91(7):1477–1487. doi:10.1017/S002531541100018X.
