# Supplementary material for: Population density and temperature correlate with long-term trends in somatic growth rates and maturation schedules of herring and sprat
Source: PLoS One. 2019 Mar 6;14(3):e0212176. doi: 10.1371/journal.pone.0212176 (PMC6402831; doi:10.1371/journal.pone.0212176)
Supplement: S2 Appendix — (PDF) [file pone.0212176.s002.pdf]

We used a continuation ratio method to produce smooth age length keys used to estimate age-length distributions [1]. This method was applied to each year of survey data separately, but we have omitted subscripts indicating year as these are not relevant to the method.

The probability that an individual is in age group a given that it is l cm long, $p_a(l)$, can be expressed as a continuation ratio

$$\theta_a(l) = \begin{cases} \frac{p_a(l)}{\sum_{i=a}^{A} p_i(l)} & a = 1, 2, \ldots, A-1 \\ 1 - \sum_{a=1}^{A-1} \theta_a(l) & a = A \end{cases} \tag{1}$$

where A is the oldest age group. The continuation ratios were parameterised as logistic curves

$$\theta_a(l) = \frac{1}{1 + e^{\alpha_a + \beta_a l}} \tag{2}$$

so that they could be calculated for each age group by estimating α_a and β_a through logistic regressions. At each length class, l, the likelihood of the modelled age distribution given the age data, $\mathbb{L}_l = \prod_{a=1}^{A} p_a(l)^{n_{a,l}}$, can be written in terms of Eq (1) as

$$\mathbb{L}_l = \prod_{a=1}^{A-1} \theta_a(l)^{n_{a,l}} (1 - \theta_a(l))^{N_{a,l}} \tag{3}$$

where $n_{a,l}$ is the number of fish of length l and age a, and $N_{a,l} = \sum_{i=a+1}^{A} n_{i,l}$. The total likelihood, $\mathbb{L} = \prod_{l=1}^{L} \mathbb{L}_l$, can be expressed in terms of Eq (3) as

$$\mathbb{L} = \prod_{l=1}^{L} \mathbb{L}_l = \prod_{a=1}^{A-1} \prod_{l=1}^{L} \theta_a(l)^{n_{a,l}} (1 - \theta_a(l))^{N_{a,l}} = \prod_{a=1}^{A-1} \mathbb{L}_a \tag{4}$$

where L is the maximum length and $\mathbb{L}_a$ is the likelihood of observing the length distribution of the age group a fish. Maximising Eq (4) with respect to α_a and β_a produces estimates of $\theta_a(l)$. Age-length distributions, the probability of age given

length, can now be derived from Eq (1).

$$p_a(l) = \begin{cases} \theta_1(l) & a = 1 \\ \theta_a(l) \prod_{i=1}^{a-1} (1 - \theta_i(l)) & a = 2, \ldots, A-1 \\ \prod_{i=1}^{A-1} (1 - \theta_i(l)) & a = A \end{cases} \tag{5}$$

The age-length distributions, derived from the survey age data, were used to estimate total sampled numbers at age and length, $M_{a,l}$, by assigning an age group to each individual present in the survey length data. The $M_{a,l}$ matrix was calculated as

$$M_{a,l} = P_{a,l}\, f_l \tag{6}$$

where $P_{a,l}$ is $p_a(l)$ written as an $A \times L$ matrix, and f_l is a column vector specifying sampled numbers at length. These $M_{a,l}$ matrices represent all of the fish present in the length data samples and the age group assigned to each individual.

References

1. Stari T, Preedy KF, McKenzie E, Gurney WSC, Heath MR, Kunzlik PA, et al. Smooth age length keys: Observations and implications for data collection on North Sea haddock. Fisheries Research. 2010;105(1):2–12. doi:10.1016/j.fishres.2010.02.004.
