# Supplementary material for: Population density and temperature correlate with long-term trends in somatic growth rates and maturation schedules of herring and sprat
Source: PLoS One. 2019 Mar 6;14(3):e0212176. doi: 10.1371/journal.pone.0212176 (PMC6402831; doi:10.1371/journal.pone.0212176)
Supplement: S3 Appendix — (PDF) [file pone.0212176.s003.pdf]

The age-length data, $M_{a,l}$, were used to generate time series of von Bertalanffy growth parameters. These were estimated separately for individual cohorts using a maximum likelihood method [1]. The mean length at age of each cohort was assumed to follow a von Bertalanffy growth curve

$$\begin{aligned} l_{i,c} &= L_{\infty\,c} - (L_{\infty\,c} - L_{0\,c}) e^{-k_c\, a_{i,c}} + \epsilon_{i,c} \\ &= \mu(a_{i,c}) + \epsilon_{i,c} \end{aligned} \tag{1}$$

where the c subscripts indicate cohort; $l_{i,c}$ and $a_{i,c}$ denote the length and age of individual i from cohort c; $L_{\infty\,c}$, k_c, and $L_{0\,c}$ are the asymptotic length, the von Bertalanffy growth rate, and length at age zero respectively; and $\epsilon_{i,c}$ are independent $\mathcal{N}(0, \sigma_c^2)$ distributed errors. A sum of squares function was defined as

$$\mathcal{S}(L_{\infty\,c}, k_c, L_{0\,c}) = \sum_i \frac{n_{a_i,c}}{v_{a_i,c}} (\bar{l}_{a_i,c} - \mu(a_{i,c}))^2 \tag{2}$$

where $n_{a_i,c}$ is the number of fish aged a_i in cohort c, and $\bar{l}_{a_i,c}$ and $v_{a_i,c}$ are the sample mean length and sample variance of length of fish aged a_i in cohort c. The log-likelihood, omitting the constant term, can now be written as

$$\mathcal{L}(L_{\infty\,c}, k_c, L_{0\,c}, \sigma_c^2) = -\frac{1}{2} N_c \ln(\sigma_c^2) - \frac{\mathcal{S}(L_{\infty\,c}, k_c, L_{0\,c})}{2\,\sigma_c^2} \tag{3}$$

where N_c is the total number of sampled fish. The maximum likelihood estimator for the error variance is given by

$$\hat{\sigma}_c^2 = \mathcal{S}(\hat{L}_{\infty\,c}, \hat{k}_c, \hat{L}_{0\,c}) / N_c \tag{4}$$

where hatted terms indicate the fitted parameters. The von Bertalanffy growth parameters were estimated separately for each cohort through numerical optimisations minimising the negative log-likelihoods.

References

1. Kimura DK. Likelihood methods for the von Bertalanffy growth curve. Fishery Bulletin. 1980;77(4):765–776.
