# Supplementary material for: Population density and temperature correlate with long-term trends in somatic growth rates and maturation schedules of herring and sprat
Source: PLoS One. 2019 Mar 6;14(3):e0212176. doi: 10.1371/journal.pone.0212176 (PMC6402831; doi:10.1371/journal.pone.0212176)
Supplement: S4 Appendix — (PDF) [file pone.0212176.s004.pdf]

The probability of an individual transitioning from juvenile to adult and becoming mature, $m(a, l, c)$, given that it is l cm long and belongs to age group a and cohort c was approximated as

$$m(a, l, c) = \frac{p(a, l, c) - p(a-1, l-\Delta l, c)}{1 - p(a-1, l-\Delta l, c)} \quad (1)$$

where $p(a, l, c)$ is the probability of being mature and Δl is the average annual growth increment [1]. Eq (1) specifies a PMRN for each cohort; that is the probability of first maturation for each combination of age and length. Linear models were used to estimate $\Delta l = l(a, c) - l(a-1, c)$ and $p(a, l, c)$.

$$l(a, c) = \beta_a + \beta_c \quad (2)$$

$$\text{logit}(p(a, l, c)) = \beta_{0,c} + \beta_1\, l + \beta_2\, a + \beta_{3,c}\, l + \beta_{4,c}\, a + \beta_5\, l\, a + \beta_{6,c}\, l\, a \quad (3)$$

Eq (2) treated cohort and age as categorical variables; Eq (3) treated cohort as a categorical variable. When using Eq (3) to model the probability of being of mature, it was rarely possible to fit the full model with all of the interaction terms. Non-significant terms were removed until the best fitting and most parsimonious models were determined. Time series of each cohort's PMRN midpoints, $L_{p50\,c}$, were estimated separately for each age group as

$$\begin{aligned} \text{logit}(m(l, c)) &= \beta_{0,c} + \beta_1\, l \\ L_{p50\,c} &= \frac{-\beta_{0,c}}{\beta_1} \end{aligned} \quad (4)$$

where cohort was treated as a categorical variable.

References

1. Barot S, Heino M, O'Brien L, Dieckmann U. Estimating reaction norms for age and size at maturation when age at first reproduction is unknown. Evolutionary Ecology Research. 2004;6(5):659–678.
